# Vitamin D Inadequacy Affects Skeletal Muscle Index and Physical Performance in Lumbar Disc Degeneration

**DOI:** 10.3390/ijms24043152

**Published:** 2023-02-05

**Authors:** Sinsuda Dechsupa, Wicharn Yingsakmongkol, Worawat Limthongkul, Weerasak Singhatanadgige, Manassanan Jitjumnong, Sittisak Honsawek

**Affiliations:** 1 Center of Excellence in Osteoarthritis and Musculoskeleton, Department of Biochemistry, Faculty of Medicine, Chulalongkorn University, King Chulalongkorn Memorial Hospital, Thai Red Cross Society, Bangkok 10330, Thailand; 2 Center of Excellence in Biomechanics and Innovative Spine Surgery, Department of Orthopaedics, Faculty of Medicine, Chulalongkorn University, King Chulalongkorn Memorial Hospital, Thai Red Cross Society, Bangkok 10330, Thailand

**Keywords:** 25(OH)D, insufficiency, lumbar disc degeneration, physical performance, vitamin D status

## Abstract

Lumbar disc degeneration (LDD) is one of the fundamental causes of low back pain. The aims of this study were to determine serum 25-hydroxyvitamin D (25(OH)D) levels and physical performance and to investigate the relationship between serum vitamin D levels, muscle strength and physical activity in elderly patients with LDD. The participants were 200 LDD patients, including 155 females and 45 males aged 60 years and over. Data on body mass index and body composition were collected. Serum 25(OH)D and parathyroid hormone levels were measured. Serum 25(OH)D was classified into the insufficiency group: <30 ng/mL and the sufficiency group: ≥30 ng/mL. Muscle strength was assessed by grip strength, and physical performance (short physical performance battery) was evaluated by the balance test, chair stand test, gait speed, and Timed Up and Go (TUG) test. Serum 25(OH)D levels in LDD patients with vitamin D insufficiency were significantly lower than in those with vitamin D sufficiency (*p* < 0.0001). LDD patients with vitamin D insufficiency had a prolonged time in physical performance on gait speed (*p* = 0.008), chair stand test (*p* = 0.013), and TUG test (*p* = 0.014) compared to those with vitamin D sufficiency. Additionally, we found that serum 25(OH)D levels were significantly correlated with gait speed (*r* = −0.153, *p* = 0.03) and TUG test (*r* = −0.168, *p* = 0.017) in LDD patients. No significant associations with serum 25(OH)D status were observed for grip strength and balance tests among patients. These findings demonstrate that higher serum 25(OH)D levels are associated with better physical performance in LDD patients.

## 1. Introduction

Low back pain is the common cause of acute or chronic musculoskeletal problems in elderly people globally. It is estimated that more than 80% of adults have experienced low back pain in their lifetime [1]. Lumbar disc degeneration (LDD) is one of the fundamental causes of low back pain. Clinically, symptoms of LDD are low back pain, difficulty walking, and impaired musculoskeletal muscle, leading to severe pain and decreased quality of life [2,3]. Several multifactorial factors contributing to the pathogenesis of LDD include mechanical stress, obesity, aging, previous injury, and genetic factors. In addition, one of the most investigated possible causes of impaired functional performance is vitamin D inadequacy. Recent reports unveiled that low vitamin D levels have been associated with functional disability, the severity of musculoskeletal pain, and poor physical performance [4,5].

Vitamin D is a lipid-soluble vitamin crucial for efficient calcium absorption. Ultraviolet radiation is essential for the production of vitamin D in the skin and is a natural source of vitamin D. Other sources of vitamin D include dietary intake, vitamin D supplements, and regular physical activity. Both indoor and outdoor physical activities enhance vitamin D levels in blood circulation [6]. Additionally, physical activity has been demonstrated to upregulate vitamin D receptor (VDR) expression and raise vitamin D blood levels [7]. The prevalence of vitamin D deficiency increases significantly with age, approximately 70% in older Asian and Caucasian populations [8]. Vitamin D and parathyroid hormone (PTH) are necessary for the maintenance of calcium homeostasis [9]. Furthermore, over 70% of degenerative lumbar spinal stenosis patients were found to have low vitamin D levels [10].

The function of vitamin D on skeletal muscle has been intensively investigated. Low serum vitamin D status has been linked to low muscle mass and poor physical performance in elderly individuals living in the community [11]. The expression of human skeletal muscle precursor cells is directly affected by the binding of 1,25-dihydroxyvitamin D (1,25(OH)D) or calcitriol, which is the active form of vitamin D and the vitamin D receptor (VDR) [12]. In vitro study has shown that the nucleus pulposus cellular metabolism is controlled by 1,25(OH)D or calcitriol, which is in parts of types I and II collagen formation [13]. In addition, Abboud et al. have shown that high levels of vitamin D maintained serum 25-hydroxyvitamin D (25(OH)D) concentration in skeletal muscle cells [14]. Therefore, vitamin D insufficiency and deficiency may play a crucial role in the pathogenesis of LDD.

According to the information above, the association between vitamin D inadequacy and musculoskeletal functionality among elderly patients with LDD needs to be further explored. In the present study, we postulated that muscle strength, physical performance, and quality of life would decrease, whereas fat mass and the percentage of fat mass would increase in LDD patients with low vitamin D concentrations. Therefore, the purposes of this study were to determine body compositions, muscle strength, and physical performance in LDD patients with vitamin D insufficiency compared to those with vitamin D sufficiency and investigate the association between vitamin D levels and body compositions, muscle strength, and physical activity in elderly LDD patients.

## 2. Results

### 2.1. Characteristics of Participants with Vitamin D Sufficiency and Insufficiency

Table 1 demonstrates a comparison between the baseline characteristics of LDD patients with vitamin D sufficiency and insufficiency. In this cross-sectional study, we recruited a total of 200 participants with LDD, who were assigned into two groups according to serum 25(OH)D levels: (i) a group of 125 patients with vitamin D sufficiency and (ii) a group of 75 patients with vitamin D insufficiency. The mean ages in the two study groups were not significantly different. We observed significant differences between the groups’ serum 25(OH)D concentrations. The median of the vitamin D sufficiency group’s 25(OH)D serum concentration was 41.2 (interquartile range, IQR: 48.0–42.8) ng/mL, and in the vitamin D insufficiency group, the median serum 25(OH)D concentration was 24.50 (24.75–22.70) ng/mL (*p* < 0.0001).

Visual analog scale (VAS), Oswestry Disability Index (ODI), and EuroQol-5 dimensions-5 level (EQ-5D-5L) scores did not differ between the two groups. However, patients with vitamin D insufficiency had a significantly higher percentage of fat mass and PTH concentration than those with vitamin D sufficiency (*p* = 0.034 and *p* = 0.027, respectively). Furthermore, the vitamin D sufficiency group had a substantially higher skeletal muscle index (SMI) than the vitamin D insufficiency group (*p* = 0.030). However, no differences in the appendicular skeletal muscle mass index (ASM) were found between the two groups.

### 2.2. Relationship between Vitamin D Levels, Body Compositions, and PTH in LDD Patients

**Figure 1:**
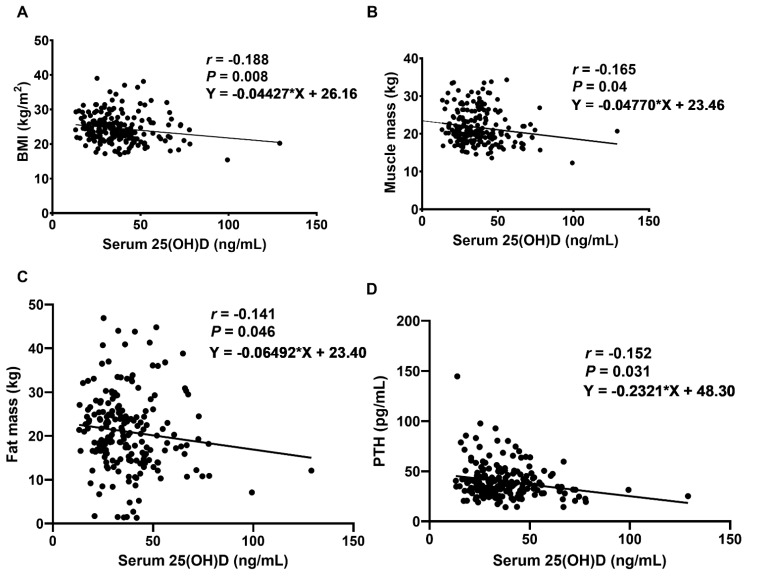
Correlations between serum 25(OH)D concentration and (**A**) BMI, (**B**) muscle mass, (**C**) fat mass, and (**D**) PTH levels in LDD patients. *r*: Spearman’s coefficient, statistically significant at *p* < 0.05.

As presented in Table 2, serum 25(OH)D levels were negatively correlated with body mass index (BMI) (*p* = 0.008, 95% confidence interval (CI) (−0.322 to −0.046)), muscle mass (*p* = 0.040, 95% CI (−0.294 to −0.015)), and fat mass (*p* = 0.046, 95% CI (−0.278 to 0.002)) in LDD patients (Figure 1). However, there was no correlation between serum 25(OH)D levels, percentage of fat mass, appendicular skeletal mass (ASM), and SMI. Furthermore, there was a negative correlation between serum levels of vitamin D and PTH levels (*p* = 0.031, 95% CI (−0.289 to −0.010)).

### 2.3. Effect of Vitamin D Levels on Muscle Strength and Physical Performance

Table 3 demonstrates the comparison of muscle strength and physical performance between LDD patients with vitamin D sufficiency and insufficiency. There were no significant differences in grip strength and balance tests.

**Figure 2:**
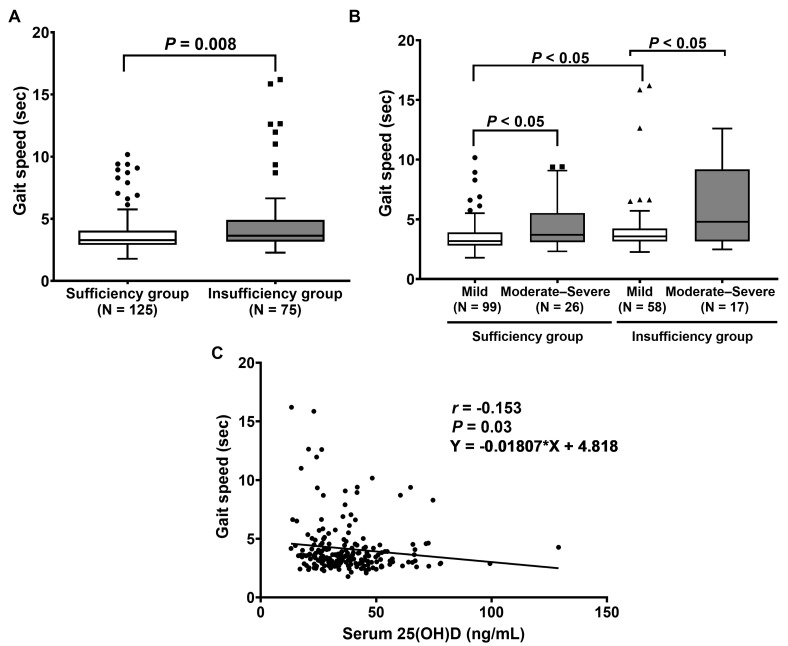
Evaluation of gait speed physical performance: (**A**) Comparison of gait speed between patients with vitamin D sufficiency and insufficiency. *p*-value was calculated with the Mann–Whitney U test. (**B**) Comparison of gait speed between patients, classified by ODI score. *p*-value was evaluated by Friedman’s two-way analysis of variance (ANOVA) test. (**C**) Correlation between serum 25(OH)D and gait speed.

Subsequent analysis revealed that the gait speed test, five-time chair stand test, and Timed Up and Go test (TUG) were significantly reduced in patients with vitamin D sufficiency. The median gait speed in patients with vitamin D sufficiency was significantly lower than in those with vitamin D insufficiency. The time in gait speed was significantly higher in the vitamin D insufficiency group than in the sufficiency group (*p* = 0.008), as shown in Figure 2A. Subsequently, the LDD patients were assigned to two groups: mild disability (0–40%) and moderate–severe disability (41–100%), according to the ODI score. The results demonstrate that the vitamin D sufficiency group with mild disability had a significantly lower time in gait speed than that with moderate–severe disability (*p* < 0.05). We found similar results between the insufficiency group with mild disability compared to that with moderate–severe disability (*p* < 0.05). Moreover, the vitamin D sufficiency group with mild disability had a significantly decreased time in the gait speed test compared to the vitamin D insufficiency group with mild disability (*p* < 0.05) (Figure 2B). Additionally, an association between serum 25(OH)D concentration and gait speed was observed. The results illustrate that serum 25(OH)D concentration was negatively correlated with gait speed (*r* = −0.153, *p* = 0.03, 95% CI (−0.282 to −0.002)) (Figure 2C).

**Figure 3:**
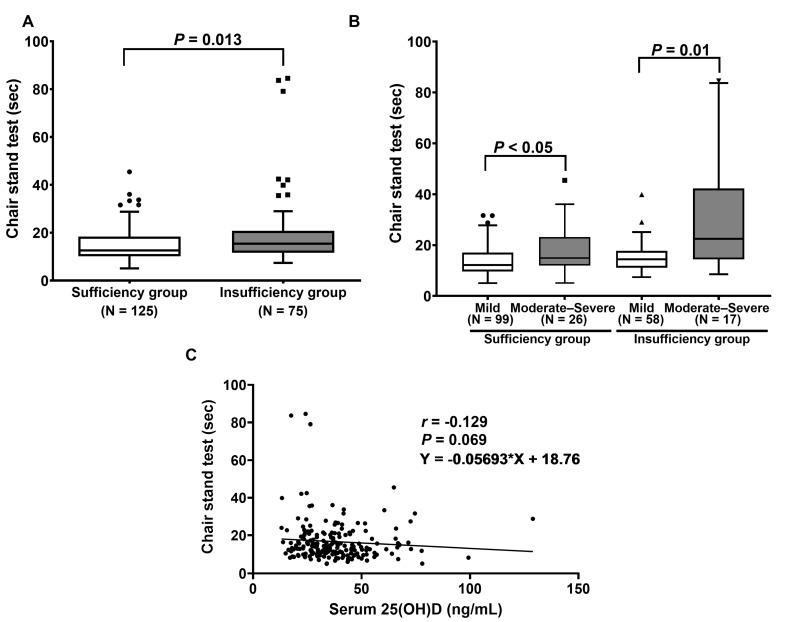
Determination of chair stand test: (**A**) Comparison of chair stand test between patients with vitamin D sufficiency and insufficiency. *p*-value was calculated with the Mann–Whitney U test. (**B**) Comparison of chair stand test between patients, classified by ODI score. *p*-value was examined using Friedman’s two-way ANOVA test. (**C**) Correlation between serum 25(OH)D and the time in chair stand test.

As displayed in Figure 3A, the scores of the five-time chair stand test were significantly higher in patients with vitamin D insufficiency than those with vitamin D sufficiency (*p* = 0.013). Regarding the ODI score, the median scores of the chair stand test in sufficiency and insufficiency patients with mild and moderate–severe disabilities were 12.2 (15.3–12.8) s, 15.0 (22.4–14.4) s, 14.4 (17.0–13.9) s, and 22.5 (46.6–20.0) s, respectively (Figure 3B). The results reveal that the vitamin D sufficiency group with moderate–severe disability had a significantly higher time in the chair stand test when compared to the group with mild disability (*p* < 0.05). We found similar results between the insufficiency group with moderate–severe disability and mild disability (*p* = 0.01). (Figure 3B). However, there was no association between serum 25(OH)D levels and the time in the chair stand test (*p* = 0.069, 95% CI (−0.267 to 0.014)), as illustrated in Figure 3C.

**Figure 4:**
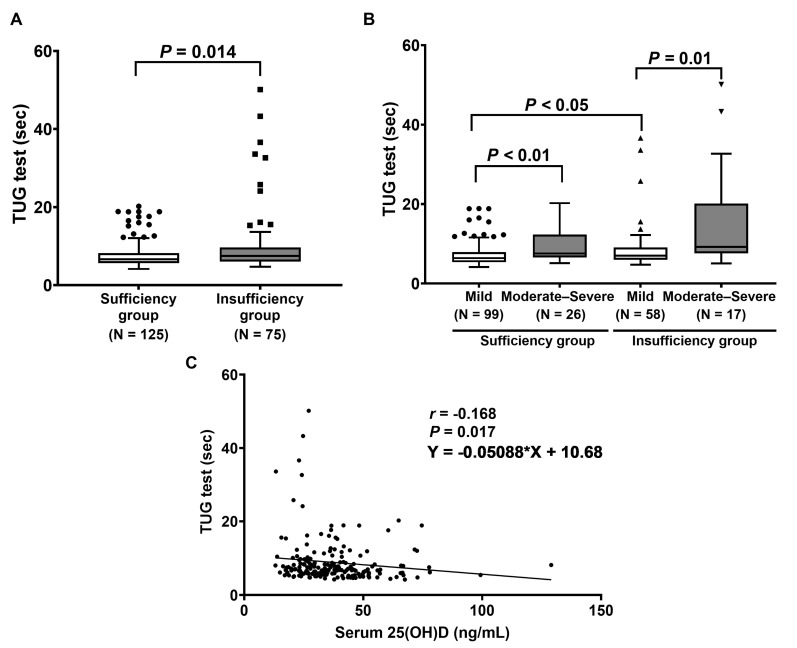
Determination of TUG test: (**A**) Comparison of TUG test between patients with vitamin D sufficiency and insufficiency. *p*-value was analyzed using the Mann–Whitney U test. (**B**) Comparison of TUG test between patients, classified by ODI score. *p*-value was calculated with Friedman’s two-way ANOVA test. (**C**) Correlation between serum 25(OH)D and the time in the TUG test.

The physical performance of the TUG test was further determined in LDD patients with vitamin D sufficiency and insufficiency. The results show that the median time in the TUG test in the vitamin D insufficiency group was also markedly greater than in the vitamin D sufficiency group (6.6 (8.5–7.2) s vs. 7.5 (12.4–8.3) s, *p* = 0.014), as displayed in Figure 4A. According to ODI disability, LDD patients with moderate–severe disability had a significantly higher time in the TUG test when compared to those with mild disability (*p* < 0.01 and *p* = 0.01, Figure 4B). Moreover, the vitamin D insufficiency group with mild disability had a significantly increased time in the TUG test when compared to the vitamin D sufficiency group with mild disability (*p* < 0.05) (Figure 4B). Additionally, serum 25(OH)D levels were inversely correlated with the time in the TUG test (*r* = −0.168, *p* = 0.017, 95% CI (−0.304 to −0.026)) (Figure 4C).

## 3. Discussion

In total, 37.5% of LDD patients had a serum 25(OH)D status below <30 ng/mL. Data analysis of this cross-sectional study suggests some outcomes of physical performance, and LDD patients with a low serum vitamin D status had significantly declined physical performance. We also observed that serum 25(OH)D levels were negatively correlated with BMI, muscle mass, fat mass, and PTH but were not correlated with ASM and SMI. Contrary to our expectations, muscle strength did not differ between the two groups.

Our findings reveal that serum PTH levels significantly increased in the vitamin D insufficiency group compared to the sufficiency group. Consistent with our results, Amphansap and coworkers reported that osteoporotic hip fracture patients with vitamin D inadequacy (insufficiency and deficiency) had higher PTH levels than those with vitamin D sufficiency [15]. According to a previous study, low vitamin D status correlated with bone turnover by enhancing PTH levels, which resulted in fall, fracture, and frailty [16]. Pro-osteoclasts are stimulated by PTH activation to develop into mature osteoclasts, which accelerate bone turnover [17]. Hence, a high vitamin D status may prevent the risk of bone fracture, fall, and impaired activity.

It is generally regarded that low vitamin D levels could promote muscular atrophy through activating particular pathways that promote protein synthesis. An increasing body of evidence proposes that the anabolic and catabolic pathways of vitamin D are involved in skeletal muscle. The imbalance of protein synthesis and degradation rates is one of the causes of muscle weakness and wasting. Bhat and coworkers documented that vitamin D deficiency in rat muscle had a high level of E2-ubiquitin conjugate enzyme and ubiquitin conjugate. Moreover, muscle atrophy F-box protein and muscle ring finger protein have been shown to exhibit a twofold increase in rat muscles with vitamin D deficiency, responding to muscle atrophy progression [18]. In addition, an increase in oxidative stress levels is associated with skeletal muscle loss. A recent study on patients with osteoarthritis showed that vitamin D deficiency increased the levels of protein carbonyl, which is a biomarker of oxidative stress and damage. It provides cellular and muscle dysfunction [19]. Moreover, Dzik et al. studied the effect of oxidative stress on multifidus muscle in patients with low back pain who had vitamin D deficiency. They reported that paraspinal muscle tissue from patients with vitamin D deficiency was related to the high levels of protein carbonyl contents, as compared to those with vitamin D sufficiency [20]. Nonetheless, the pathological mechanisms of vitamin D on muscle function need to be explored further.

Regarding muscle strength and physical performance, we found that LDD patients with vitamin D sufficiency had significantly enhanced clinical outcomes of physical performances, including the gait speed test, chair stand test, and TUG test but did not exhibit changes in grip strength and balance tests compared to those with vitamin D insufficiency. According to previous studies, inadequate vitamin D administration and low levels of vitamin D both increase the incidence of low back pain [21,22]. In this aspect, our results are consistent with the findings of several previous reports. Bislev et al. reported that the TUG test results were significantly improved in older women with vitamin D sufficiency [23]. Moreover, Uchiyama and coworkers have shown better physical performances, such as chair stand time, functional reach, and grip strength, among community-dwelling men with vitamin D sufficiency. It was postulated that an increase in serum 25(OH)D was associated with better physical performance [24]. A possible explanation for these discrepancies may include the low levels of serum 25(OH)D and VDR expression. It has long been accepted that the active form of vitamin D or 1,25(OH)D plays a momentous role in skeletal function, which regulates the mitogen-activated protein kinase signaling pathway and other gene expressions. According to the vitamin D levels and VDR expression, a high level of 1,25(OH)D concentration enhances the level of VDR expression. Once 1,25(OH)D treatment interacts with the VDR ligand, it increases the activation of VDR expression and might stimulate the protein synthesis of skeletal muscle and prevent type 2 muscle fiber atrophy [25,26]. Therefore, it may be assumed that optimal vitamin D status has the potential to improve muscle strength and physical performance in patients with vitamin D inadequacy.

In regard to the association between vitamin D levels and muscle strength, physical performance, and other parameters, the results demonstrate a significant negative correlation between gait speed, TUG test, BMI, muscle mass, fat mass, and PTH. In this aspect, our findings are in line with the findings of several previous studies. Tieland et al. highlighted that low serum 25(OH)D status appeared to be associated with impaired physical performance, including the chair rise and gait speed tests, in a group of community-dwelling elderly people [11]. Iolascon et al. reported that serum vitamin D status was negatively correlated with the SPPB walking speed test and the chair stand test in postmenopausal women [27]. Moreover, a previous study revealed a negative association between circulating 25(OH)D status and chair stand time in old Japanese men [24]. A possible explanation for the correlation between serum 25(OH)D and physical performance might be attributed to the imperative role of 1,25(OH)D or calcitriol, which promotes mitochondrial biogenesis in skeletal muscle cells [28] and controls calcium levels via regulating the function of calcium pumps and modulating muscle contraction and relaxation [25]. Sufficient circulating vitamin D can result in enhancing and prolonging physical ability. However, a recent study evinced that serum 25(OH)D levels were not related to muscle mass and strength [29]. Welford and coworkers found that circulating vitamin D status was not correlated with appendicular skeletal muscle mass in postmenopausal women [29]. These conflicting findings may be ascribed to differences in populations, physical activity, various seasons of sample collection, and/or the measurements applied.

This study has several inevitable limitations. First, the present study did not include a healthy control group without the condition LDD. Second, our study was based on a relatively small number of LDD patients, especially the vitamin D insufficiency group. Therefore, this could limit the statistical power of our findings and generalizability. Third, we did not examine the general physical activity levels, which could also influence vitamin D deficiency. Lastly, this study was designed as a cross-sectional study; thus, the cause-and-effect relationships could not be established. A prospective longitudinal study is warranted to identify the exact role of vitamin D in LDD patients.

In conclusion, serum 25(OH)D levels were negatively correlated with BMI, muscle mass, fat mass, TUG time, and gait speed in LDD patients. Our findings highlight that LDD patients with vitamin D sufficiency have better physical performance and physical function compared to those with low vitamin D status. Therefore, we recommend that LDD patients should receive vitamin D supplementation for improving muscle strength, quality of life, and physical performance.

## 4. Materials and Methods

### 4.1. Participants

This cross-sectional study was conducted at the outpatient clinic of the Department of Orthopaedics at King Chulalongkorn Memorial Hospital during November 2021–June 2022. Two hundred patients with LDD agreed to participate. All participants were investigated by interviewing about medical disease history. The demographic characteristics of patients were obtained from their medical records. The inclusion criteria included patients with lower back pain and symptomatic radiculopathy to LDD. The exclusion criteria included history of spine surgery, primary hyperparathyroidism, neurological conditions (i.e., Parkinson’s disease, previous stroke), previous epidural steroid injection, or disabilities that prevented physical activity. Participants were also excluded if they received vitamin D3 analogs, anticonvulsants, or anti-tuberculosis medications.

The study protocol was approved by the Institutional Review Board of the Faculty of Medicine at Chulalongkorn University (IRB approval no. 427/65). Written informed consent was obtained from all individual participants prior to entering the study.

### 4.2. Visual Analog Scale and Oswestry Disability Index Assessment

Patients were evaluated for pain level by visual analog scale (VAS) score evaluation instrument. The score is displayed as 0–10 ordinal scale, with a higher score indicating a higher level of pain. Pain and functional disability were assessed using Oswestry Disability Index (ODI), Thai version, which is recommended for spinal disorder measurements [30]. This questionnaire has 10 items, including pain, personal care, lifting, walking, sitting, standing, sleeping, sex life, social life, and traveling. Each one has 6 statements and each statement is scored from 0 to 5 points [30]. Total score was scored out of 100. In this evaluation, the ODI scores were categorized into 2 groups, including mild disability (0–40%) and moderate–severe disability (41–100%). In addition, the EuroQol-5 dimensions-5 level (EQ-5D-5L) scores were evaluated across five dimensions, including mobility, self-care, usual activities, pain/discomfort, and anxiety/depression. The questionnaire is scored at five levels, from no problems to extreme problems [4].

### 4.3. Anthropometric and Body Composition Measurements

Height, weight, and waist circumference (WC) were determined using standard measurement procedures. Body mass index (BMI) was calculated by dividing weight (kg) by the square of height (m^2^). Appendicular skeletal mass (ASM), percentage of fat mass, and fat mass were assessed using bioelectrical impedance analysis (BIA) (BC-418 Segmental Body Composition Analyzer; Tanita Corporation, Tokyo, Japan). ASM was estimated as the sum of skeletal muscle mass of the arms and legs in kilograms. Skeletal muscle index (SMI) was calculated as percentage of ASM divided by body weight (%).

### 4.4. Muscle Strength and Physical Performance

Muscle strength and physical performance were measured by physical therapists. Grip strength was assessed by handgrip dynamometer (kilograms) (Takei Scientific Instruments Co., Ltd., Tokyo, Japan), which was evaluated as an index of muscle strength in the upper limbs. Short physical performance battery (SPPB) score was measured to evaluate physical performance. It consists of 3 tests, including gait speed tests (4 m), standing balance test, and chair stand test. The first test was the 4 m gait speed test, which measured the time needed to walk 4 m (s) twice and calculated for average time. The second test was the chair stand test. Participants folded their arms across their chest and rose from the chair as fast as possible 5 times. The time was measured in seconds from initial to final position. The last test was the balance test, which evaluated the body balance with three types of standing. Participants stood without the use of a walker or cane: (i) a side-by-side stand (stood with feet together), (ii) semi-tandem stand (stood with the side of the heel of one foot touching the big toe of the other foot), and (iii) tandem stand (stood with the heel of one foot in front of and touching the toe of the other foot), each for 10 s. Each test was scored from 0 to 4 points. A higher score indicates better physical performance (0–12 points) [31]. The Timed Up and Go test (TUG) measured the time needed to stand up from a chair, walk 3 m, return to a chair, and sit down (s).

### 4.5. Serum and Plasma Preparation

Fasted early morning venous blood was collected from patients and centrifuged at 4000 rpm for 10 min, with serum and plasma samples. Serum levels of 25(OH)D were analyzed using chemiluminescent immunoassay (DiaSorin, Inc., Stillwater, MN, USA). LDD patients were classified into two groups: vitamin D insufficiency group at <30 ng/mL and vitamin D sufficiency group at ≥30 ng/mL. Parathyroid hormone (PTH) was determined by electrochemiluminescence method (Roche Diagnostics GmbH, Mannheim, Germany).

### 4.6. Statistical Analysis

Data were analyzed using the Statistics Package for Social Science (SPSS software) version 22.0 for Windows (SPSS, Inc., Chicago, IL, USA). Figures were established using GraphPad Prism version 9.0. The demographic category data of LDD patients between sufficiency and insufficiency groups were analyzed by chi-squared test, unpaired Student’s *t*-test, and Mann–Whitney U test where appropriate. Friedman’s two-way analysis of variance was employed for the comparison of continuous variables among LDD subgroups. The data were expressed as n (%), mean ± standard deviation (SD), or median and interquartile ranges (IQRs). Spearman’s rank correlation coefficient test was used to calculate the association between vitamin D levels, body composition, muscle strength, and physical performance. A *p*-value < 0.05 was considered to be statistically significant for differences and correlations.

## Figures and Tables

**Table 1:** Demographic data of LDD patients with vitamin D sufficiency and insufficiency.

Variables	Vitamin D Status		*p*-Value
	Sufficiency Group (n = 125)	Insufficiency Group (n = 75)	
Age (years)	66.1 ± 9.3	66.2 ± 9.6	0.366 ^b^
Gender, n (%) Female Male	98 (78.4) 27 (21.6)	57 (76.0) 18 (24.0)	0.728 ^a^
Body composition BMI (kg/m^2^) Muscle mass (kg) Percentage of fat mass (%) Fat mass (kg) ASM (kg) SMI (%)	24.2 ± 4.3 20.3 (22.4–20.7) 32.6 ± 11.0 20.5 ± 9.3 16.0 (17.2–16.3) 28.2 ± 4.3	25.0 ± 3.8 21.1 (22.9–20.9) 34.3 ± 8.9 21.7 ± 7.7 16.0 (17.4–16.3) 27.3 ± 3.3	0.388 ^b^ 0.424 ^c^ **0.034**^b^ 0.067 ^b^ 0.649 ^c^ **0.030**^b^
Serum 25(OH)D (ng/mL)	41.2 (48.0–42.8)	24.5 (24.7–22.7)	**<0.0001** ^c^
PTH (pg/mL)	37.5 ± 14.1	43.1 ± 20.5	**0.027** ^b^
VAS (0–10)	5.0 (6.0–5.0)	6.0 (6.0–4.7)	0.991 ^c^
Oswestry Disability Index (ODI)	30.3 ± 12.1	30.5 ± 14.2	0.147 ^b^
Oswestry Disability Index (ODI) Mild disability, n (%) Moderate disability, n (%) Severe disability, n (%)	99 (79.2) 25 (20.0) 1 (0.8)	58 (77.3) 16 (21.3) 1 (1.3)	0.907 ^a^
EQ-5D-5L	0.7 (0.7–0.6)	0.7 (0.7–0.6)	0.611 ^c^

Values are reported as means ± standard deviation (SD) or medians (IQR). Significant results are shown in bold. ASM: appendicular skeletal muscle mass index; SMI: skeletal muscle index; 25(OH)D: 25-hydroxyvitamin D; PTH: parathyroid hormone; VAS: visual analog scale; ODI: Oswestry Disability Index; and EQ-5D-5L: EuroQol-5 dimensions-5 level. *p*-value < 0.05 insufficiency vs. sufficiency. ^a^ Chi-square test. ^b^ Unpaired Student’s *t*-test. ^c^ Mann–Whitney U test.

**Table 2:** Correlations between serum 25(OH)D and different parameters (n = 200).

Variables	*r*	*p*-Value	95% CI
Age (years)	0.085	0.233	(−0.060 to 0.236)
BMI (kg/m^2^)	−0.188	**0.008**	(−0.332 to −0.048)
Muscle mass (kg)	−0.157	**0.026**	(−0.281 to −0.008)
Percent of fat mass (%)	−0.056	0.429	(−0.168 to 0.087)
Fat mass (kg)	−0.141	**0.046**	(−0.263 to −0.008)
ASM (kg)	−0.115	0.105	(−0.250 to 0.012)
SMI (%)	0.131	0.065	(−0.009 to 0.262)
VAS	0.024	0.738	(−0.119 to 0.165)
ODI	0.013	0.853	(−0.145 to 0.160)
EQ-5D-5L	0.012	0.861	(−0.131 to 0.154)
PTH (pg/mL)	−0.125	**0.031**	(−0.315 to −0.007)
Grip strength (kg)	−0.058	0.417	(−0.187 to 0.080)
Balance test (points)	0.041	0.566	(−0.089 to 0.180)
Gait speed (s)	−0.153	**0.030**	(−0.013 to −0.287)
Chair stand test (s)	−0.129	0.069	(−0.260 to 0.014)
Timed Up and Go test (s)	−0.168	**0.017**	(−0.331 to −0.016)
Short physical performance battery (SPPB) (points)	0.118	0.095	(−0.022 to 0.271)

*r*: Spearman’s coefficient; statistically significant at *p* < 0.05.

**Table 3:** Muscle strength and physical performance in LDD patients according to vitamin D levels.

Variables	Vitamin D Status		*p*-Value
	Sufficiency Group (n = 125)	Insufficiency Group (n = 75)	
Grip strength Dominant (kg)	18.9 (21.5–19.1)	19.1 (22.2–18.8)	0.829
Physical performance Balance test (points) Gait speed (s) Chair stand test (s) Timed Up and Go test (s)	4.0 (3.8–3.5) 3.3 (4.1–3.5) 13.4 (17.9–14.4) 6.6 (8.5–7.2)	4.0 (3.6–3.0) 3.7 (5.9–4.1) 15.4 (22.9–16.0) 7.5 (12.4–8.3)	0.056 **0.008** **0.013** **0.014**
SPPB (points)	10.0 (10.3–9.7)	10.0 (9.6–8.3)	**0.011**

Data are reported as medians (IQR). Significant results are shown in bold. Results were determined using the Mann–Whitney U test. *p*-value < 0.05 insufficiency vs. sufficiency.

## Data Availability

The data presented in this study are available on request from the corresponding authors.

## Abbreviations

25(OH)D	25-hydroxyvitamin D
1,25(OH)D	1,25-dihydroxyvitamin D
ASM	Appendicular skeletal mass
BIA	Bioelectrical impedance analysis
BMI	Body mass index
EQ-5D-5L	EuroQol-5 dimensions-5 level
IQR	Interquartile range
LDD	Lumbar disc degeneration
ODI	Oswestry Disability Index
PTH	Parathyroid hormone
SMI	Skeletal muscle index
SPPB	Short physical performance battery
SPSS	Statistical Package for the Social Sciences
TUG	Timed Up and Go
VAS	Visual analog scale
VDR	Vitamin D receptor
WC	Waist circumference
